# Uniform Suspension of the Clustered Triamcinolone Acetonide Particle

**DOI:** 10.1155/2013/315658

**Published:** 2013-01-28

**Authors:** Masahiko Sugimoto, Mineo Kondo, Masayuki Horiguchi

**Affiliations:** ^1^Department of Ophthalmology, Mie University, School of Medicine, 2-174 Edobashi, Mie, Tsu 514-8507, Japan; ^2^Department of Ophthalmology, Fujita Health University, 1-98 Dengakugakubo, Kutsukake-cho, Toyoake, Aichi. 470-1192, Japan

## Abstract

*Purpose*. MaQaid (MaQ) is a new triamcinolone acetonide commercialised in Japan to visualize the vitreous. Because MaQ is preservative-free, it has a lower risk of ocular toxicities. However, since MaQ is only available as a powder, it needs suspenssion. Suspension does not always result uniformally, which causes poor visibility. This study reports a new MaQ suspension for better visibility. *Methods*. After medium addition to a MaQ vial, various methods were used. These included the use of (1) vortex mixer, (2) two syringes and a three-way stopcock, and (3) ultrasonic washer. We calculated suspended MaQ concentration (*n* = 5). To evaluate the reproducibility, we estimated the coefficient of variance (CV, *n* = 3). We used this MaQ for pig eyes, and vitreous visualization was simulated. Subsequently, we used this MaQ suspension for humans. *Results*. MaQ suspensions were sucessfull, and the concentrations of single particles increased significantly (*P* < 0.01). The CV was 36.1% for the routine method and 9.03% ffor the new method. Administration of a suspended MaQ made it possible to clearly visualize the vitreous in both pig and human eyes. *Conclusions*. We devised new techniques for uniformal MaQ suspension. These new methods can compensate for the MaQ disadvantages and ensure a safety surgery.

## 1. Introduction

 MaQaid (MaQ, Wakamoto Pharmaceutical Co., Ltd., Tokyo, Japan) is a new triamcinolone acetonide (TA) agent that has been marketed in Japan since the end of 2010. Compared to the other TA agents (e.g., Kenalog and Kenacort, Bristol-Myers Squibb, New York, NY, USA), MaQ has several advantages: (1) it has been approved for use as a means for intraoperative visualization of the vitreous by the Ministry of Health, Labour and Welfare of Japan, and (2) it is preservative-free. Like other preservative-free TA agents currently being marketed in the USA(Triamcinolone Acetonide PF, New England Compounding Center, Framingham, MA, USA), this compound was designed solely for ophthalmological use. In addition, it has been shown that this agent has a lower risk for ocular toxicity, such as the preservative-induced sterile endophthalmitis.

**Figure 1:**
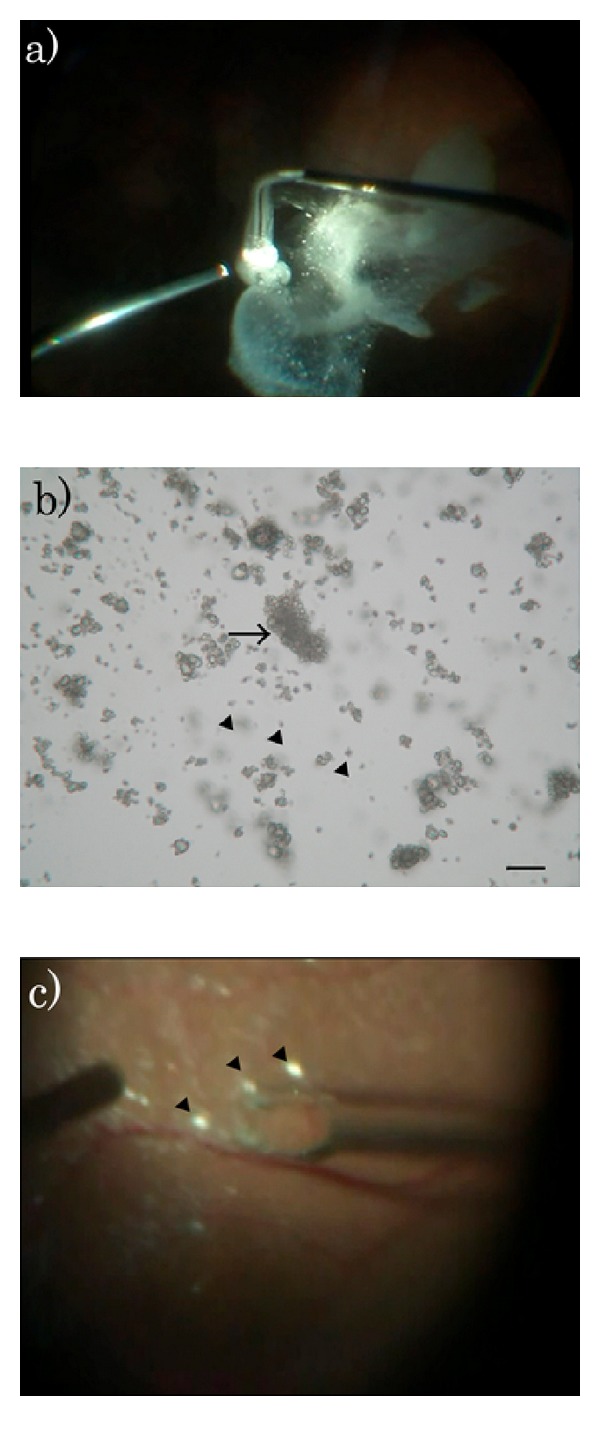
Occurrence of nonuniform clustered MaQ particles when using the routine suspension method. MaQ was suspended using the routine protocol recommended by the manufacturer. The case is epiretinal membrane. During surgery, clustered MaQ spread nonuniformly in the vitreous (a). Under microscope, nonuniform clustered particles are seen (b, arrow). Note only small numbers of single particles are seen (arrowheads). TA mass disturbs visibility during retina surface managements (c). Clustered MaQ disturbed membrane peeling. Bar: 50 *μ*m.

Since MaQ is only available in the powdered form, it needs to be suspended prior to use. The supplied data sheet recommends that dissolved MaQ is placed into a syringe and further shaken manually in order to facilitate a homogeneous suspension. However, a homogenous suspension of single particles is often difficult if not impossible to achieve when using this method (Figure 1). Moreover, suitable preparation of a homogeneous suspension for use in surgical procedures requires specific skills, which makes it difficult for routine medical staff or assisting physicians to successfully perform. As such, the nonuniform clustered particles that result can decrease the visibility during surgical procedures [1]. In addition, the remaining clustered particles can also cause various postoperative complications. These complications are similar to those associated with excess remaining steroid and include cataracts, intraocular hypertension, and sterile endophthalmitis.

This study established new ready-to-hand methods that could be performed by using available devices commonly found in hospitals. With these new methods, a MaQ suspension to single particles can quickly and easily be prepared prior to surgery. In addition, these new suspension methods markedly improved the intraoperative visibility and simplified the removal of the particles. This paper discusses the development and use of our new methods for simplifying the MaQ preparation.

## 2. Materials and Methods

### 2.1. Materials

 MaQ (40 mg/vial) was purchased from Wakamoto Pharmaceutical Co., Ltd. (Tokyo, Japan), while Opeguard MA was purchased from Senju Pharmaceutical Co., Ltd., (Osaka, Japan). MaQ was mixed with 4 mL of OpeguardMA to achieve a final concentration of 10 mg/mL. The clustered particles were suspended using the various methods described below in an attempt to yield homogeneous suspensions.

### 2.2. Homogeneous Suspensions of Clustered MaQ

#### 2.2.1. Method 1: Suspension Using: A Vortex Mixer

**Figure 2:**
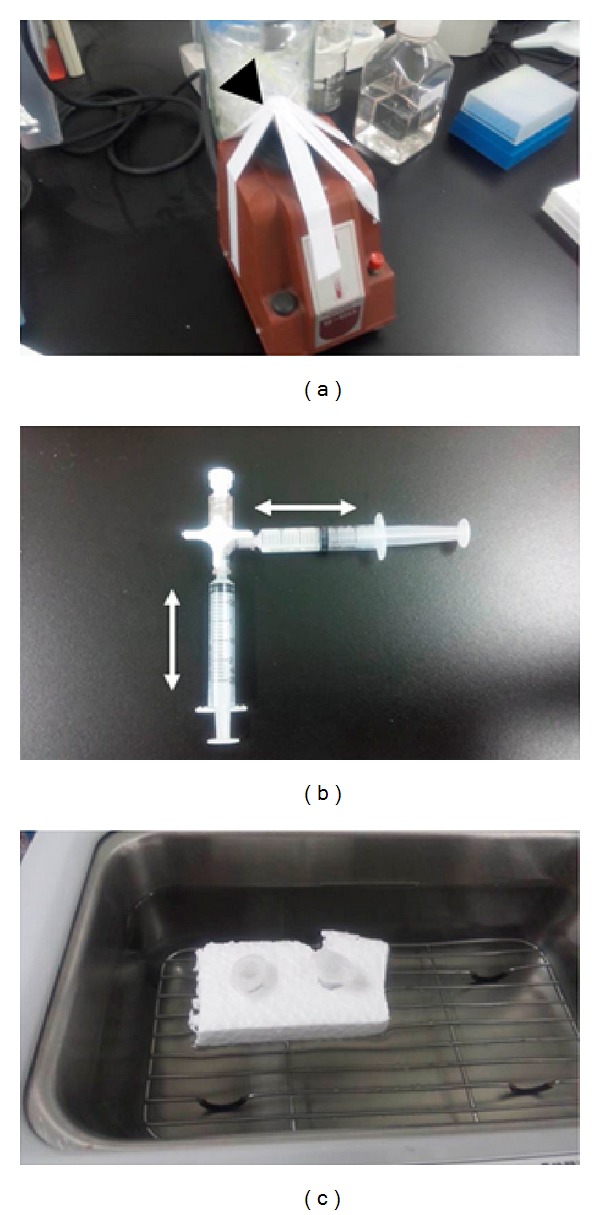
New suspensions methods using various instruments. Vortex mixer used in the experiments is shown (a). The arrowhead points to the site that vibrates, and for each suspension, the tube was fixed on the mixer using vinyl tape (arrow). Syringe and three-way stopcock circuit suspensions system is shown (b). Samples are first placed within one of the syringes. Processing is then performed by repeatedly pushing the sample between the two perpendicularly arranged syringes. Fluid travels via the three-way stopcock, with movement occurring as the directional arrows. Ultrasound washer suspension is shown (c). Tubes are mounted on a styrofoam float and placed within the water tank. Ultrasound vibrations cause suspension of the samples while they are floating on top of the water.

 The mixture (1 mL) was placed in a 1.5 mL tube. After fixing the tube in place on a vortex mixer (S-50, Taitec, Koshigaya, Japan) and securing it with vinyl tape, the mixture was agitated for 15 or 30 min (Figure 2(a)). 

#### 2.2.2. Method 2: Suspension Using a Circuit Composed of a Syringe and a Three-Way Stopcock

 The mixture (2 mL) was placed in a 2.5 mL syringe (Nipro Syringe 2.5 mL Luer Chip, Medical Device Certification No. 220AABZX00244000, Nipro Medical Corporation, Osaka, Japan). The circuit setup is shown in Figure 2(b) and was prepared using an additional syringe connected to a three-way stopcock (BD Connecta, REF 394906, Becton Dickinson Infusion Therapy AB, Helsingborg, Sweden). The suspension was repeatedly passed within this circuit for 5 or 10 min (Figure 2(b), arrows). 

#### 2.2.3. Method 3: Suspension Using an Ultrasonic Washer

 The mixture (1 mL) was placed in a 1.5 mL tube. The top was sealed with paraffin film and then mounted on a float made of styrofoam, as shown in Figure 2(c). The tube was then agitated for 15 or 30 min within the water-filled ultrasonic washer (OY-3N001, As One Corporation, Osaka, Japan). 

### 2.3. Counting of Suspended Single MaQ Particles

Prepared samples were observed under a microscope. Successful suspensions were defined based on the concentration of the number of small particles. The number of suspended single particles per unit area was then counted, with a blood cell counting chamber used as an indicator of the homogenous suspension. Briefly, this procedure required taking each sample (15 *μ*L) and dropping it into the blood cell counting chamber, after which the number of suspended single particles within the reference grid was counted (*n* = 5/method). A nonrepeated ANOVA test was employed for the intergroup comparisons.

### 2.4. Evaluation of the Coefficient of Variance

 To evaluate reproducibility of the suspension method, we compared the coefficient of variance (CV) of the routine suspension method recommended by the manufacturer with the syringe/three-way stopcock suspension method (*n* = 3). The CV between the operator to operator and between the ocassion to occasion were evaluated. After counting the suspended single particles, we then estimated the average and standard deviation. The CV was estimated from the following formula:

(1) CV=standard  deviationaverage  ×100  (%).

### 2.5. Administration in Pig Eyes

Pig eyes were used for vitrectomies and conduction of vitreous visualization simulations. After each suspended MaQ sample (0.2 mL/eye) was injected into the vitreous, the kinetics were then observed under a surgical microscope (S5, Carl Zeiss, Oberkochen, Germany).

### 2.6. Preparation for Human Vitrectomy

All procedures were approved by the Ethics Review Committee of the Mie University Hospital. After adding 40 mg of MaQ to 4 mL OpeguardMA, we then used the syringe/three-way stopcock suspension method at a clean operation area to create the MaQ suspension of single particles. After performing core vitrectomy, we then injected 0.2 mL of the prepared MaQ suspension mixture. The kinetics of MaQ were observed during the surgical procedure.

## 3. Results

### 3.1. Single Particle Counts to Compare the Homogenous Suspensions

**Figure 3:**
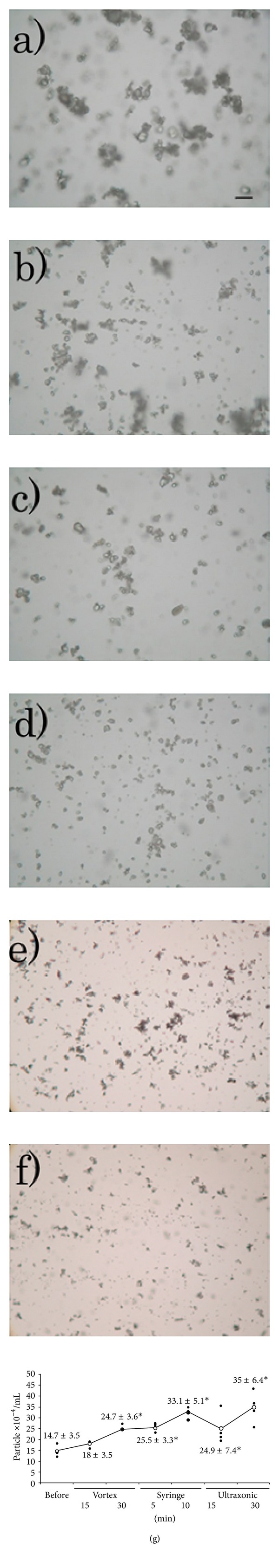
Microscopic images of the suspended homogenate particles. Microscopic images of the MaQ particles are shown after suspensions using the different techniques. All methods yielded homogeneous suspensions. Vortex mixer used for 15 min (a) or 30 min (b). Syringe system used for 5 min (c) or 10 min (d). Ultrasonic washer used for 15 min (e) or 30 min (f). Single particle counts for the homogenate particle suspensions is shown (g). Processing by each technqiue resulted in significant increases in the separated particle counts as compared to the preprocessing counts. *P < 0.01, nonrepeated ANOVA. Bar: 50 *μ*m.

 Smaller suspended MaQ particles and a better suspension were obtained for each of the new methods as compared to the manufactuer's recommended method (Figures 3(a)–3(f)).  Figure 3(g) lists the suspended single particle counts per unit area. Statistically, with the exception of the vortex mixer suspension (15 min), each of the methods had a significantly better homogeneous suspension compared to the unsuspended samples (*P* < 0.01, nonrepeated ANOVA).

### 3.2. Evaluation of the CV

 The operator-to-operator CV was 36.1% for the routine method and 9.03% for new method. The occasion-to-occasion CV was 11.73% for the routine method and 6.56% for the new method.

### 3.3. Comparison of Particle Kinetics during the Vitrectomy Simulation Model

**Figure 4:**
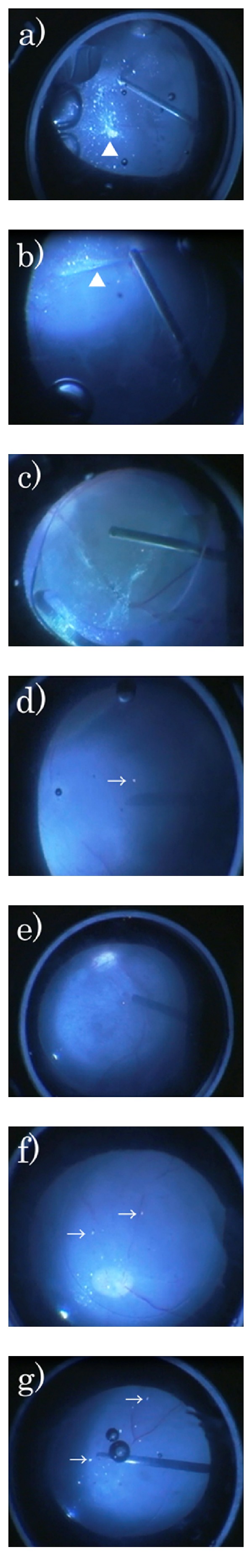
Comparison of the particle kinetics during vitreous surgery in the pig eye. Surgical images of MaQ samples (0.2 mL each) injected to the vitreous of the pig eye. Arrowheads show the clustered MaQ particles. Each of the methods led to the particles becoming smaller (arrows) and the visibility improving. Routine suspension (a). Vortex mixer used for 15 min (b) or 30 min (c). Syringe system used for 5 min (d) or 10 min (e). Ultrasonic washer used for 15 min (f) or 30 min (g).

 After injection of untreated MaQ, a large number of clustered particles were observed and scattered throughout the vitreous cavity (Figure 4(a)). When the vortex method was used, a smaller number of clustered particles were observed. In addition, as seen in Figures 4(b) and 4(c), the longer the treatment time, the greater the increase in the number of single particles observed. 

 When the samples from the syringe method were injected, an almost complete disappearance of the clustered particles was observed. This tendency became more marked following longer treatments (Figure 4(d)). When mist-form TA, which contains extremely fine particles, was scattered within the vitreous cavity after a longer treatment, a reduction in the vitreous visibility was oberved (Figure 4(e)).

 Injection of the ultrasonic washer samples caused a decrease in the number of clustered particles, resulting in an improved visibility (Figure 4(f)). However, small clusters were still seen for treatments that used 30 min ultrasonic processed samples (Figure 4(g)).

### 3.4. Particle Kinetics during Vitrectomy in Humans

**Figure 5:**
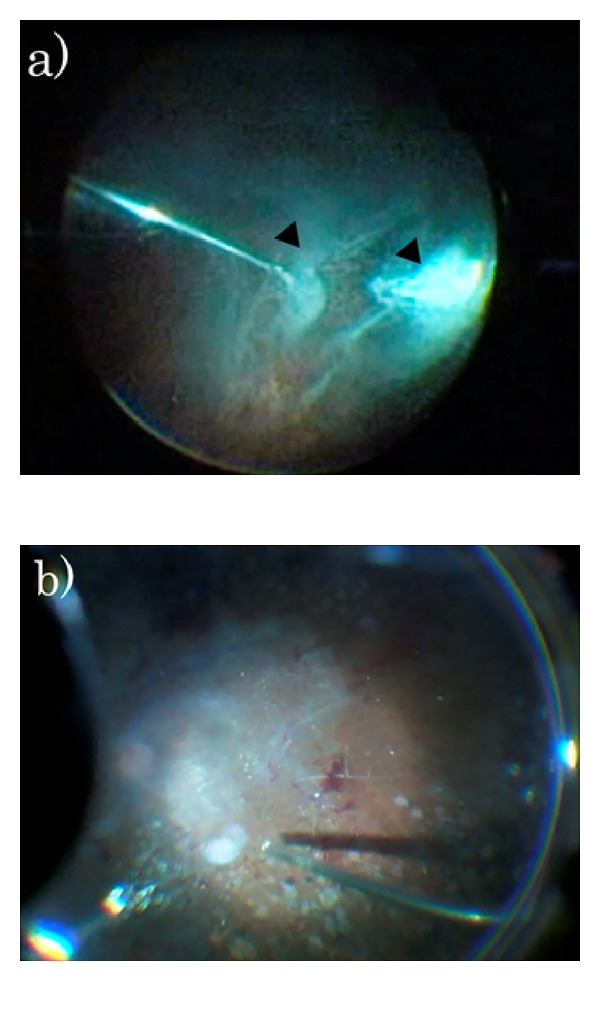
Human eye images observed when using MaQ suspensions from the syringe system. Surgical image after injection of MaQ suspensions created by the syringe system. MaQ was injected into human eyes during vitrectomies. (a) Arrowheads show the well-suspended MaQ particles, with a clearly visualized vitreous due to the decreased number of clustered particles. (b) Since clustered particles were not spread on the retinal surface, it was possible to easily peel the membrane.

 Having proved that we could successfully mix and use a MaQ suspension with both in vivo and in vitro models, we decided to use the MaQ suspension in human eyes during surgery. We show a typical case in Figure 5. The case is proliferative diabetic retinopathy. After core vitrectomy, TA was injected to visualise vitreous cortex. Administrations of well-suspended MaQ created using the syringe method made it possible to clearly visualize the vitreous without any interference due to large clustered particles (Figure 5(a)). Additionally, these particles did not cause any problems when performing retinal surface procedures (Figure 5(b)). 

## 4. Discussion

 There is a long history for the use of intraocular administrations of steroids. However, towards the end of the 1970s, many reports began to appear concerning the effects of intraocular steroid administrations used for suppressing ocular inflammation. These studies reported that these administrations could lead to a progression of proliferative vitreoretinopathy or endophthalmitis [2–4]. Subsequently, intravitreal TA injections were shown to be an effective treatment for macular edema due to diabetic retinopathy, retinal vascular disease, and uveitis [5]. Furthermore, other studies reported using TA to visualize the vitreous body during vitreous surgery [6, 7]. Better visibility during surgery helped to increase the safety of intraoperative manipulations, which reduces the incidence of complications (e.g., intraoperative retinal tears and retinal detachment) and improves the overall postoperative outcome [8, 9]. In Japan, Kenacort (Kenalog in the USA) had been the only commercialized TA available for clinical usage. Even though Kenacort had been utilized for eye treatments, this intraocular usage has not been officially authorized. Thus, Kenacort was primarily applied as an off-label use only after approval of institutional review boards. 

Unlike Kenacort, MaQ has been approved for use in intraoperative visualization of the vitreous. Thus, MaQ is clinically advantageous, as it can be used without causing ethical problems. Another marked advantage of MaQ is that it is preservative-free. It is widely known that intravitreous TA injections can sometimes cause sterile endophthalmitis, with preservatives in the mixtures thought to possibly be responsible for these occurrences. However, the actual reported incidence rate of this remains quite low (0.87 to 7.3%) [10–12]. To prevent this, it has been recommended that the preservative should be removed through the use of various methods [13–15]. Even with these methods, sterile endophthalmitis cannot be completely prevented, as it has been suggested that the preservative is not necessarily the only factor responsible for causing this inflammatory condition [16]. Additionally, even though intraocular injections of TA work quite well, ophthalmologists sometimes hesitate to inject TA due to ongoing concerns that sterile endophthalmitis could still occur. Thus, the advantage of using MaQ is that it is preservative-free. 

 Since MaQ is only available in the powdered form, it needs to be suspended in a medium prior to its use. To date, only a few reports have been published on techniques for creating a homogeneous TA suspension. While most of these previous studies did focus on creating finer Kenacort particles [15, 17, 18], unfortunately the techniques suggested were not necessarily simple and they required special devices. MaQ's manufacture is now trying to modify MaQ an easy-resolve powder. But unfortunately, it does not seem to be improved as we expected (in-house document from Wakamoto). Thus, even though it has been shown to be advantageous to use this drug, the difficulties encountered when trying to prepare the powder have limited its application clinically. The techniques reported in the present study make it possible to prepare suspensions by only using commonly available devices, thereby making it much easier to clinically apply the drug. It should also be taken into account that these methods also make it easier for the routine medical staff to prepare MaQ solutions for use during surgical procedures. 

 We tested all three preparation techniques and confirmed that each of the methods resulted in significantly homogeneous suspensions. The injection of these suspensions into the pig vitreous confirmed that the suspensions were homogenous enough for clinical use. We then evaluated the reproducibility of these MaQ suspensions. Although the CV was high for the routine preparation method that was suggested by the drug manufacturer, a further dramatic improvement was seen when using the syringe method. Since the reproducibility of the suspension is related to the preparation stability, it is important that the same conditions for the preparation must exist for each surgical procedure. Therefore, we decided to test one of our new simple preparations and determine its clinical usefulness. Based on this point, we attempted to use the syringe system to create the MaQ suspensions for the surgical procedures. Using this new method, the suspended particles made it possible to clearly visualize the vitreous. Since these results proved it was possible to overcome the difficulties in making single MaQ suspensions, we concluded that our newly developed MaQ suspension techniques could be safely utilized during surgical procedures. 

With regard to which of the three techniques would be the most useful clinically, we examined the required time period to process suspension prior to the surgeries. Suspensions using either vortex or ultrasound took a minimum of 15 min, which is an acceptable amount of time. However, with these two techniques, the samples have to be taken out of the clean operating area and processed in an unclean area. Once the particles are suspended, the samples are then returned to the clean operating area. During these steps, there is the possibility that bacterial contamination could occur. In contrast, all of the steps for the syringe technique can be conducted within the clean area, with complete suspension occurring over a markedly shorter time (5 min). Because of these advantages, we concluded that suspensions using the syringe system would be the most practical among the three different techniques.

**Figure 6:**
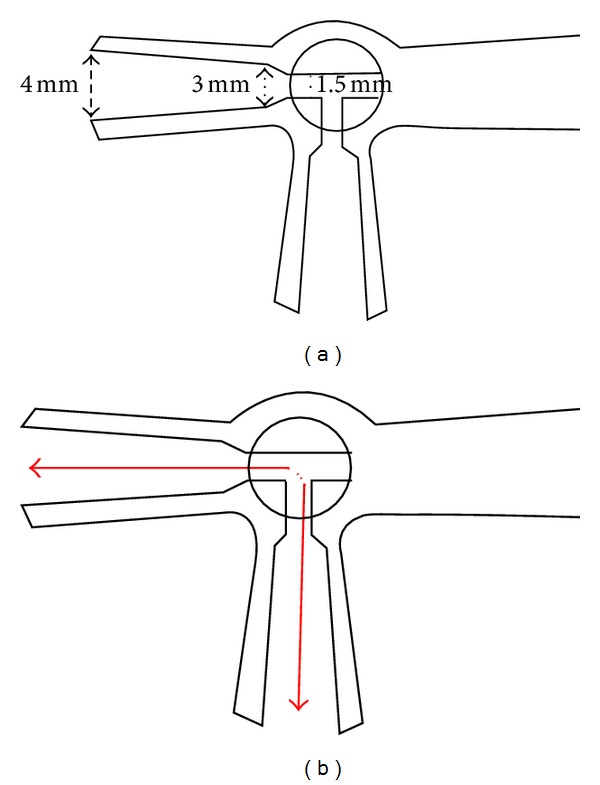
Three-way stopcock used during the suspensions. The lumen of the three-way stopcock was narrower in the central part (1.5 mm) than in the peripheral part (4 mm). (a) Fluid flows through the route marked by the arrows (b). The broken line indicates where there were sharp changes in the direction due to the particles hitting the wall during passage through the stopcock.

Among the techniques examined in this study, the vortex and the ultrasonic washer techniques were designed to suspend the particles by causing vibrations. In contrast, the syringe/three-way stopcock technique did not involve the use of vibrations. The three-way stopcock was designed to have a junction at which the inner lumen diameter differed from that of the outer lumen diameter (Figure 6(a)). The passage of fluid through such a junction causes the formation of swirls, which leads to the TA particles being exposed to forces that are different from those forces that are produced by the ordinal flow. Consequently, this force appeared to lead to a breakdown of the particle clusters, thereby leading to a suspension that only contained single particles. As seen in Figure 2(b), the circuit was assembled so that 2 syringes were arranged perpendicularly to each other (at an angle of 90 degrees), instead of in a 180-degree opposing arrangement. When fluid repeatedly passes through this type of circuit, the fluid is forced to change its direction of flow by 90 degrees (Figure 6(b)). The particles within the fluid hit against the inner wall at the point where the direction of flow changes, which we believe was ultimately responsible for the particle suspension that subsequently occurred. 

## 5. Conclusion

We report on an effective method that can be used to create a homogeneous suspension containing only single particles. This technique for MaQ is both simple and reliable and thus, helps to improve the overall safety of these surgical procedures.
